# Insights on Stereoselective Residue and Degradation of Spirotetramat Enantiomers on *Tubifex* of the Qinghai Plateau

**DOI:** 10.3390/ijms27031170

**Published:** 2026-01-23

**Authors:** Hongyu Chen, Yang Zhang, Kaifu Zheng, Shuo Shen, Shujing Yu, Wei Li

**Affiliations:** 1 Academy of Agriculture and Forestry Sciences, Qinghai University, Xining 810016, China; hongyuchenred@163.com (H.C.); yangzh-123@163.com (Y.Z.); zhengkf@qhu.edu.cn (K.Z.); wenzhanggz@163.com (S.S.); 2 Key Laboratory of Qinghai-Tibetan Plateau Biotechnology, Qinghai University, Xining 810016, China; 3 Scientific Observing and Experimental Station of Crop Pest in Xining, Ministry of Agriculture, Xining 810016, China; 4 Key Laboratory of Agricultural Integrated Pest Management of Qinghai Province, Xining 810016, China; 5 National Pesticide Engineering Research Center (Tianjin), College of Chemistry, Nankai University, Tianjin 300071, China

**Keywords:** spirotetramat, enantiomers, *Tubifex*, residue, degradation

## Abstract

This study established an HPLC-MS/MS method to quantify the enantiomers of spirotetramat in tubifex. To assess the accuracy and precision of the approach, recovery tests were conducted for insecticide. For all enantiomers, the limits of detection were 0.003 mg/kg. The quantization limits were 0.01 mg/kg. Spirotetramat enantiomers recovery rates in tubifex were found to be between 81 and 114%, with relative standard deviations being less than 7%. The half-lives of spirotetramat enantiomers in tubifex were 3.81–10.58 d, respectively. The 22.4% spirotetramat suspension was sprayed on tubifex three times at a low dosage (high dosage advised). After 14 days after harvesting, the terminal residues of spirotetramat enantiomers in the tubifex were less than 0.03 mg/kg. The findings offer a quantitative foundation for setting China’s maximum residue limits as well as a recommendation for the safe and responsible usage of spirotetramat enantiomers in tubifex.

## 1. Introduction

Qinghai Province, also referred to as “China’s Water Tower” and the “Roof of the World”, is tucked away on the northeastern edge of the Qinghai–Tibet Plateau and has an ecosystem of unmatched ecological value [1]. It is a vital hub for water conservation, biodiversity preservation, and global climate regulation because of its distinct physical location, which includes high-altitude grasslands, alpine marshes, glaciers, and lakes [2]. These functions go well beyond its administrative boundaries. Qinghai is the source of significant river systems that support billions of people throughout Asia [3,4,5]. Three of the most important rivers on the continent—the Lancang (Mekong), Yellow, and Yangtze Rivers—were born in the Qinghai Plateau, of which Qinghai is a fundamental part [6]. The Sanjiangyuan National Nature Reserve, located in southern Qinghai, is particularly crucial: it produces approximately 25% of the Yangtze River’s total runoff, 49% of the Yellow River’s, and 15% of the Lancang River’s annual water flow [7]. These rivers nourish lush plains, promote agricultural output, and supply the drinking water demands of more than 1.8 billion people in China and Southeast Asia. Qinghai is essential to the control of the world climate. As a key “carbon sink”, the province’s extensive grasslands, marshes, and permafrost store large amounts of carbon, helping to moderate global warming [8]. The Qinghai–Tibet Plateau’s cryosphere—including glaciers and permafrost—acts as a “water tower” for Asia, regulating the timing and volume of river runoff. Changes in Qinghai’s ecosystems directly affect regional and global climate patterns, including monsoon systems and precipitation distribution [9]. Additionally, the plateau’s high altitude and clean air make it a significant place for atmospheric circulation, impacting weather patterns across a large range of Asia. In recent years, China has enacted a number of tough ecological protection measures in Qinghai. The construction of Sanjiangyuan National Park, the first national park in China, has effectively protected the source areas of the three major rivers through measures such as ecological migration, grazing ban, and wetland restoration. The Qinghai Lake National Nature Reserve has successfully restored the lake’s water level and wetland area, leading to a major recovery in the population of black-necked cranes and other endangered species. Furthermore, efforts such as grassland ecological compensation and desertification management have helped to strengthen the resilience of Qinghai’s ecosystems. These initiatives not only maintain local biodiversity and ecological balance but also contribute to global ecological security and sustainable development. In summary, Qinghai’s ecological significance transcends its physical limits [10]. It is an essential part of the global ecological system because of its functions as a supply of water, a hotspot for biodiversity, and a regulator of the temperature. The continuous protection efforts in Qinghai underscore China’s commitment to ecological conservation and sustainable development, setting an example for global ecological governance. In addition to being crucial for the welfare of people in China and its neighbors, protecting Qinghai’s ecosystems is also a shared duty for preserving the planet’s ecological balance and future [11].

**Figure 1:**
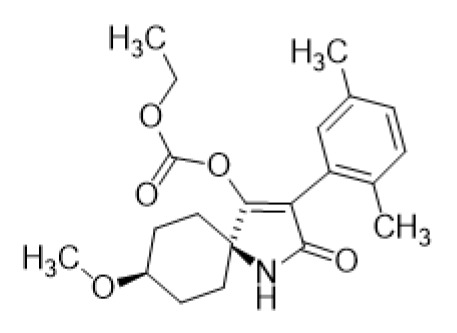
Chemical structures of spirotetramat.

Spirotetramat (Figure 1) is an insecticide known for its environmental credentials, and as such, there has been considerable research conducted on its environmental aspects [12,13,14]. This research generally focuses on its impact on non-target organisms, its persistence in the environment, and its role in integrated pest management (IPM) systems [15,16,17]. Ecotoxicology: Researchers examine the toxicity of spirotetramat to a number of non-target species, including helpful insects like pollinators, as well as fish, birds, and mammals [18,19]. These investigations are vital in verifying the safety of the substance when administered in agricultural settings [20,21]. 1. Persistence and Degradation: The breakdown of spirotetramat in the environment is researched to estimate its persistence [22,23,24]. Researchers investigate how it degrades in soil, water, and on plant surfaces under diverse environmental circumstances [25]. This is essential because the longer a pesticide lingers in the environment, the higher the potential risk to non-target species [26]. 2. Leaching and Runoff: Research looks into how spirotetramat might permeate the soil and contaminate surface or groundwater [27]. This is particularly significant in regions where water quality is a concern [28]. 3. Environmental Fate: The total environmental fate of spirotetramat is examined, which includes its diffusion, transformation, and accumulation in the environment following application [29,30]. 4. Impact on Ecosystems: Research also analyzes the effects of spirotetramat on ecosystem health, including the richness and number of organisms in agricultural and adjacent habitats [31]. Studies assess how spirotetramat works in conjunction with other pest control techniques, like biological control agents and cultural practices, to maintain a sustainable and eco-friendly approach to pest management because it is frequently employed as part of IPM strategies [32].

This article focuses on the ecological toxicity effects of spirotetramat enantiomers in the Qinghai Plateau. Therefore, we systematically compiled the half-lethal concentration (LC_50_) data for vertebrates, invertebrates, and common non-target organisms. By comparing these data with those of the target species and non-target species (such as the characteristic economic crop of the Qinghai Plateau, the Goji berry), we can better assess its toxicity. Among vertebrates, under aqueous exposure conditions, the 96h LC_50_ of striped bass (*Morone saxatilis*) was the lowest (500 μg/L), while the 48h and 96h LC_50_ values of northern cricket frog (*Acris crepitans*) were 2000 μg/L and 1700 μg/L, respectively. For zebrafish (*Danio rerio*), the 96h LC_50_ ranged from 3.07 to 7.21 mg/L, and the 48 h LC_50_ of tadpoles of Asiatic toad (*Bufo gargarizans*) was 6.98 mg/L. Under oral exposure, the 10-day LC_50_ values of 10-day-old northern bobwhite (*Colinus virginianus*) and 20-day-old Japanese quail (Coturnix japonica) reached 2117 ppm and 1327 ppm, respectively. Among invertebrates, the 96h LC_50_ of amphipods (*Gammarus* spp.) under aqueous exposure was 110 μg/L, the 48 h oral LC_50_ of Italian honeybee (*Apis mellifera ligustica*) was 6.14 mg/L, the 48h contact LC_50_ of 2nd instar nymphs of sweetpotato whitefly (*Bemisia tabaci*) was 4.0665 mg/L, while the 10-day contact LC_50_ of adults increased to 471.6547 mg/L. For newly hatched crawlers of Japanese maple scale (*Parthenolecanium corni*), the 48 h contact LC_50_ was 722.27 mg/L. Among non-target organisms, the 96h LC_50_ of silkworm (*Bombyx mori*) larvae via contaminated leaves was 11.52 mg/L (highly toxic). For Eisenia fetida, the 14-day LC_50_ under soil exposure was >1000 mg/kg dry soil; for adult earthworms, the 14-day LC_50_ under soil exposure was >100 mg/kg dry soil, and the 48 h contact LC_50_ was 550 (440–690) μg/cm^2^, all classified as low toxicity. The results indicated that spirotetramat exhibited significant differences in toxicity to different organism groups, with high safety for soil annelids. The high toxicity risk to silkworms suggests that strict control of spirotetramat use is necessary in mulberry fields and their surrounding areas [33]. At present, most of the research on the ecological toxicity of spirotetramat focuses on the parent compound of spirotetramat. However, there has been no report on the ecological toxicity of its enantiomers. This study not only improved the toxicity assessment of the target organisms, but also added the toxicity evaluation of non-target crops (including the maximum residue limit), which can provide some scientific data for ecological assessment.

Results from these investigations generally indicate that spirotetramat has a positive environmental profile with little toxicity to non-target organisms and a relatively low potential for environmental persistence [34]. It is meant to be utilized in a way that reduces the possibility of detrimental effects on the environment, which is why it is regarded as a significant tool in sustainable agriculture. However, to guarantee that environmental dangers are minimized, appropriate application methods and attention to label instructions are essential [35].

The body of spirotetramat is the subject of all current research due to the difficulty of separating its enantiomers. The enantiomers of spirotetramat have not been studied. But since our lab has separated the enantiomers of spirotetramat, our research is extremely valuable.

We created a modified HPLC-MS/MS method to investigate the dissipation and residues of spirotetramat enantiomers in tubifex. The results could help Chinese government representatives make suggestions on the safe and proper use of spirotetramat enantiomers in tubifex.

## 2. Results

### 2.1. Optimization of HPLC-MS/MS Conditions and Sample Pretreatment

As there is no mature enantiomer separation technology for spirotetramat, our laboratory has developed a basic separation technology.

The mass spectrometer was configured with the following operational parameters: The collision-induced dissociation (CID) gas pressure was kept at 230 kPa, and the atomizing and heating gas flow rates were set at 3.0 L/min and 10.0 L/min, respectively. The heating block and desolvation line (DL) temperatures were set at 450 °C and 250 °C, respectively, while the interface temperature was kept at 300 °C. Multiple reaction monitoring (MRM) was used for detection, and an electrospray ionization (ESI) source with positive ion scanning mode (ESI+) was used for ionization.

The following optimal conditions were used for the chromatographic separation: acetonitrile (phase A) and ultrapure water (phase B) made up the mobile phase. The following parameters were used in a gradient elution program: Phase A’s percentage grew linearly from 30% to 90% in the first three minutes, remained at 90% for the next four minutes, then dropped back to 30% for the next six minutes, and remained at 30% until the completion of the ten-minute run. A steady flow rate of 0.25 mL/min was used during the whole elution procedure. Using a Shimadzu LC-20AD liquid chromatography system, the separation was accomplished on a Superchiral S-AD chiral column with dimensions of 2.0 mm × 75 mm and a particle size of 2.2 μm. Furthermore, the injection volume was fixed at 1 μL and the column temperature was regulated at 40 °C.

**Figure 2:**
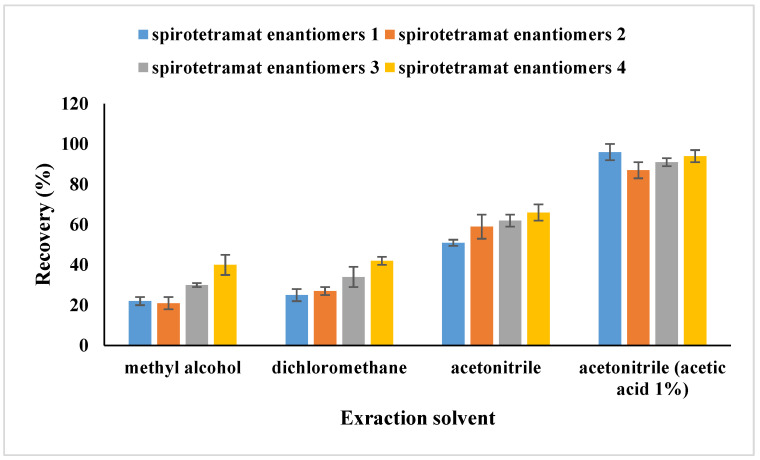
Extraction efficiency of spirotetramat enantiomers in tubifex with different extraction solvents.

**Figure 3:**
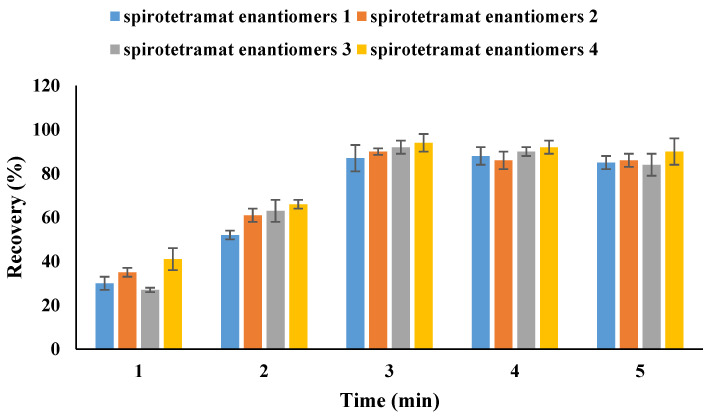
Extraction efficiency of spirotetramat enantiomers in tubifex with different extraction times.

**Figure 4:**
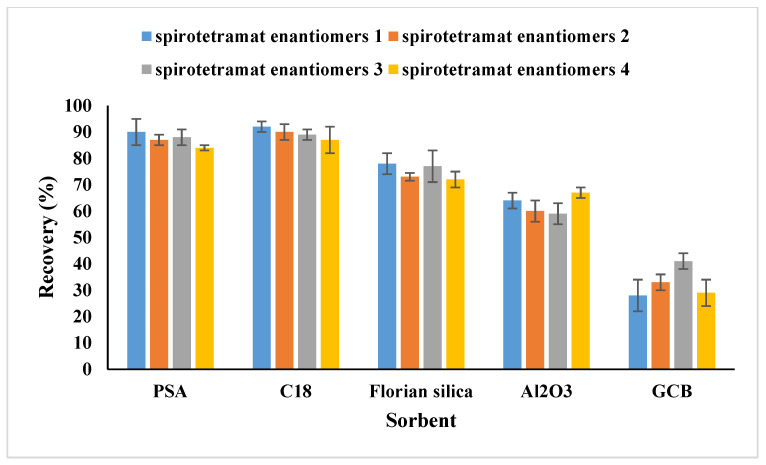
Extraction efficiency of spirotetramat enantiomers in tubifex with different adsorbents.

**Figure 5:**
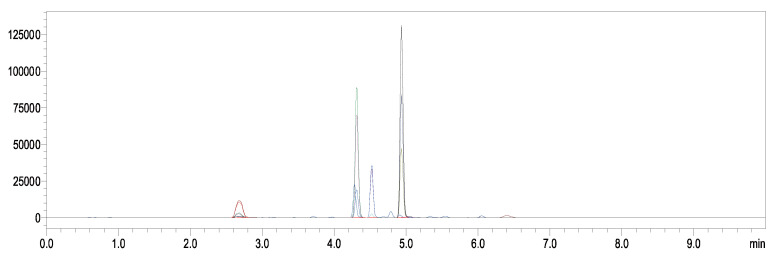
Chromatograms of spirotetramat enantiomers in tubifex.

One aim of our work is to establish a modified QuEChERS method for spirotetramat enantiomer determination in tubifex. Methanol, dichloromethane, acetonitrile, and acetonitrile containing 1% acetic acid were among the solvents that were evaluated for the extraction process based on the physicochemical properties of the target analytes. The use of acetonitrile with 1% acetic acid as the extraction solvent produced acceptable recovery rates, as shown in Figure 2. The impact of different extraction times was then examined in order to further enhance the extraction recovery of spirotetramat enantiomers from tubifex. The result (Figure 3) showed that 3 min was used as the extractive time and more satisfactory recoveries were achieved. In the present study, the cleanup efficacy of dispersive solid-phase extraction (d-SPE) using sorbents including PSA, C_18_, GCB, Al_2_O_3_, and Florisil was evaluated in comparison with the non-cleanup approach (i.e., without any sorbent addition). The evaluation was performed based on two key metrics: the analyte recovery rate and the level of matrix interference. For this purpose, 50 mg each of PSA, C_18_, GCB, Al_2_O_3_, and Florisil were introduced into 1 mL of the standard solution with a concentration of 0.5 mg/L. Following sample pretreatment and subsequent instrumental analysis, the recovery rates of spirotetramat enantiomers were summarized and presented in Figure 4. The results showed that spirotetramat enantiomers were absorbed by GCB, and the ability of AL_2_O_3_ and Florian silica to remove impurities in tubifex is poor. As a result, the tubifex samples were extracted using acetonitrile containing 1% acetic acid, and then they were purified using a composite sorbent system that contained 50 mg of PSA and 50 mg of C18. The advantages of simplicity, time efficiency, and environmental friendliness are demonstrated by this well-established pretreatment technique for the extraction and purification of spirotetramat enantiomers from tubifex. Figure 5 shows representative chromatograms of the two target enantiomers in the standard solution. All spirotetramat enantiomers met the criteria for qualitative and quantitative analysis, as shown in the figure, with perfect baseline separation, symmetric and crisp peak morphologies, and suitable retention time windows. The results showed that the cleanup phase was well suited and that the approach was highly sensitive.

### 2.2. Method Validation

The suggested approach was validated using a traditional verification methodology, including linearity, limits of quantification, limits of detection, matrix effects, accuracy, and precision among the criteria that were assessed.

External matrix-matched standards of spirotetramat enantiomers were used to evaluate the method’s linearity. Seven concentration gradients (0.05, 0.2, 0.5, 1, 2, 5, and 10 mg/kg) were set for each matrix separately. Positive linear connections were found for spirotetramat enantiomers, and all relevant correlation coefficients were more than 0.999. The LOQ was defined as the lowest spiking concentration with a relative standard deviation (RSD) of less than 20% that may produce acceptable recovery rates between 70% and 110%. In the meantime, the concentration of matrix-matched standards at which the analyte signal strength tripled the level of background noise was known as the LOD.

Matrix effects (% ME) were calculated using the equation.

ME = (m_matrix_ − m_solvent_)/m_solvent_ × 100%

Here, m_matrix_ and m_solvent_ stand for the slopes of calibration curves found in the matrix and pure solvent systems, respectively, while ME stands for the matrix effect. A ME value of zero indicates that there is no matrix interference with the analyte; a positive value indicates that the matrix can improve the analyte’s reaction, while a negative value indicates that the matrix inhibits the analyte’s response. The testing results showed that the tubifex matrix may improve the reactivity of spirotetramat enantiomers, as shown in Table 1.

Recovery studies were conducted on spirotetramat enantiomers to evaluate the precision and accuracy of the suggested approach. In particular, the target enantiomers were added to blank tubifex samples at three different concentrations (0.05, 1.0, and 5.0 mg/kg). The findings showed that spirotetramat enantiomer recovery rates in tubifex varied from 81% to 112%, with corresponding relative standard deviations (RSDs) of 2.01% to 5.97%. These results further confirmed that the recently developed analytical method had acceptable reproducibility and satisfactory performance for the identification of spirotetramat enantiomers in tubifex, as shown in Table 2.

### 2.3. Dissipation Dynamics

A first-order kinetic equation (C_t_ = C_0_e^−kt^) was adopted to establish the pesticide dissipation equation. In this formula, C_t_ refers to the residual concentration of the pesticide at time t, C_0_ indicates the initial concentration, and k represents the rate constant expressed in day^−1^. Additionally, the half-life (t_1/2_) of each experiment was calculated from the obtained k value using the formula t_1/2_ = ln2/k.

In 2024, the dynamic test of degradation in the tubifex of Qinghai was carried out. The dosage was 667 times liquid, applied once. The degradation curves of spirotetramat enantiomers in tubifex were C = 0.3150e^−0.0655t^ (spirotetramat enantiomers 1), C = 0.4773e^−0.1772t^ (spirotetramat enantiomers 2), C = 0.3756e^−0.1821t^ (spirotetramat enantiomers 3) and C = 0.4823e^−0.0758t^ (spirotetramat enantiomers 4). The degradation half-lives were 10.58 d (spirotetramat enantiomers 1), 3.90 d (spirotetramat enantiomers 2), 3.81 d (spirotetramat enantiomers 3) and 9.14 d (spirotetramat enantiomers 4) (Table 3).

The dynamic test of tubifex degradation in spirotetramat enantiomers in 2024 showed that the dosage was 667 times liquid. The degradation rate of spirotetramat enantiomers in tubifex in four spirotetramat enantiomers exceeded 50% after 3 and 7 days, and the degradation rate reached 90.77% (spirotetramat enantiomers 1), 85.19% (spirotetramat enantiomers 4) and 100.00% (spirotetramat enantiomers 2, 3) after 28 days of application, respectively.

### 2.4. Terminal Residues

The final residue test on tubifex in spirotetramat enantiomers in 2024 included 1000-fold liquid at low dose and 667-fold liquid at high dose, applied 3 and 4 times, and sampled 3, 5 and 7 days after the last application interval. The final residue of 7 d in spirotetramat enantiomers 1 was 0.12~0.25 mg/kg, and the final residue of 7 d in spirotetramat enantiomers 2 was 0.00~0.04 mg/kg. The final residue of 7 d in spirotetramat enantiomers 3 was 0.00~0.03 mg/kg, and the final residue of 7 d in spirotetramat enantiomers 4 was 0.23~0.30 mg/kg. (Table 4).

## 3. Discussion

Spirotetramat enantiomers, as an endogenous quaternary keto acid insecticide, are widely used due to their remarkable effect in controlling piercing-sucking pests. However, it can seep into aquatic environments through farmland runoff and soil infiltration, leaving pesticide residues and posing a potential threat to aquatic organisms. The tubifex is a key freshwater benthic organism, serving as both a carrier of pollutant accumulation and an environmental indicator species. Exploring the degradation pattern and residue dynamics of Spirotetramat enantiomers in its body is of great significance for assessing the ecological risk of pesticide residues in water bodies and improving the theory of pesticide environmental fate.

The degradation dynamics of spirotetramat enantiomer residues are related to the ecological safety of aquatic organisms. Normal degradation can reduce residues in the body and decrease the inhibition of the growth and reproduction of tubifex. When degradation is blocked, residues and highly toxic metabolites accumulate, causing abnormal movement and reproduction of chatters. Long-term low-dose exposure reduces egg-laying by 20% to 30%, posing a threat to the population. Moreover, the residues in the body of the tremors are transferred through the food chain and accumulate as the trophic level increases, triggering a biological amplification effect, damaging the nervous and reproductive systems of high-trophic organisms, and disrupting the structure of the food chain and ecological stability. The metabolic residues excreted by it entering the water body may also alter the microbial community and affect the material cycle. The long-term effects remain to be studied. 

Future research needs to combine the characteristics of pesticide residues, analyze the toxicity thresholds and transformation rules of different metabolic residues, and clarify the accumulation mechanism of high-risk residues. It should also explore the role of intestinal flora in residue degradation and discover efficient degradation functional flora, as well as build a multi-media and multi-trophic level residue exposure model to simulate the migration and transformation paths of residues in natural water bodies, providing a basis for formulating pesticide use standards, controlling water body residue pollution, and protecting aquatic ecology.

## 4. Materials and Methods

### 4.1. Apparatus

The experimental apparatus included a QL-901 vortex mixer (Haimen Qilinbeier Instrument Manufacturing Co., Ltd., Haimen, China) and a TGL-20B centrifuge (Shanghai Anting Scientific Instrument Factory, Shanghai, China). Meanwhile, spirotetramat enantiomers were determined using a Shimadzu HPLC-MS/MS-8040 instrument fitted with a Superchiral S-AD chromatographic column purchased from Restek Corporation (Bellefonte, PA, USA).

### 4.2. Chemicals

Spirotetramat enantiomer standards were independently prepared in our laboratory. A 22.4% spirotetramat suspension, the test formulation, was furnished by Shanxi Xiannong Biotechnology Co., Ltd. (Weinan, China). Solvents including acetonitrile, n-hexane and acetic acid were acquired from Tianjin Baishi Chemical Industry Co., Ltd. (Tianjin, China). Analytical-grade chemicals such as activated anhydrous magnesium sulfate, sodium chloride and sodium acetate were procured from Tianjin Guangfu Technology Development Co., Ltd. (Tianjin, China). Additionally, primary secondary amine (PSA, 40–60 μm particle size), octadecylsilane (C18, 40–60 μm particle size) and graphitized carbon black (GCB, 30–90 μm particle size) were obtained from Agela Technologies (Tianjin, China).

### 4.3. Analytical Methods

#### 4.3.1. Sample Preparation

After the application of spirotetramat enantiomers, a 300 g representative tubifex sample was randomly collected from each treatment plot at different intervals (2 h, 1, 3, 5, 7, 10, 14, 21, and 28 days). In order to perform residue detection, tubifex specimens were also gathered from each terminal residue field [36]. Prior to further investigation, all collected samples were kept in a deep freezer at −20 °C.

#### 4.3.2. Extraction and Cleanup

A 10.0 g portion of homogenized tubifex was accurately weighed into a 50 mL centrifuge tube, followed by the addition of 20 mL acetonitrile containing 1% acetic acid. The resulting mixture was intensely vortexed for 2 min, and 1.5 g of sodium acetate was subsequently added. Immediately after adding sodium acetate, the solution was vortex-mixed for 30 s and then subjected to centrifugation at 6000 rpm for 3 min. The acetonitrile fraction was passed through anhydrous magnesium sulfate for dehydration; a 10 mL aliquot of the filtrate was collected and concentrated to near dryness at 45 °C under reduced pressure. Sample purification was achieved via dispersive solid-phase extraction: the dried residue was reconstituted in 1 mL of n-hexane and transferred to a 2 mL centrifuge tube loaded with 50 mg of PSA and 50 mg of C18. After vortexing for 30 s, the tube was centrifuged at 10,000 rpm for 3 min. Finally, the supernatant was filtered through a 0.45-μm nylon membrane filter (Millipore, Billerica, MA, USA) prior to HPLC-MS/MS analysis.

#### 4.3.3. HPLC-MS/MS

Collision-induced dissociation (CID) gas pressure was set at 230 kPa; the flow rates of atomizing gas and heating gas were configured to 3.0 L/min and 10.0 L/min, respectively. The interface, desolvation line (DL), and heating block were maintained at temperatures of 300 °C, 250 °C, and 450 °C. Electrospray ionization in positive mode (ESI+) was adopted as the ion source, and the detection was performed using multiple reaction monitoring (MRM) mode.

The gradient elution program was programmed as follows: 30–90% of phase A within 0–3 min, maintained at 90% A for 3–4 min, reverted to 30% A over 4–6 min, and held at 30% A from 6 to 10 min. The separation was performed on a Shimadzu LC-20AD liquid chromatography system, with a Superchiral S-AD chromatographic column (2.0 mm × 75 mm, 2.2 μm) as the stationary phase. The column temperature was set at 40 °C, the injection volume was fixed at 1 μL, and a constant flow rate of 0.25 mL/min was applied throughout the entire elution process.

### 4.4. Field Experiments

Since this pesticide is widely used in agricultural fields, part of our experiment involves simulating field tests and planning ecological experiments.

To investigate dissipation dynamics of spirotetramat enantiomers in tubifex, field experiments were carried out with an area of 20 m^2^. The spirotetramat (SC, 22.4%) was dissolved in water and sprayed at an active constituent dose of 667 times liquid (1.5 times higher than the recommended high dosage) on the surface of soil (tubifex). In total, 10 L water was applied to dissolve 6.67 mL spirotetramat (SC, 20%) for each experimental plot. Representative soil samples (including tubifex) were randomly taken from each experimental plot for the dissipation investigation at two hours after spraying (the initial concentration), as well as one, two, three, five, seven, ten, fourteen, twenty-one, and twenty-eight days following pesticide application.

A 30 m^2^ spirotetramat-free control plot was prepared concurrently with two treatment groups that each had three 20 m^2^ replicate plots for inter-treatment separation in order to perform the terminal residue experiment in soil (tubifex). Four regimens were used to apply the 22.4% spirotetramat SC: low dosage (1000-fold dilution, recommended high dose) with three or four applications, and high dose (667-fold dilution, 1.5× recommended high dose) with three or four applications, with a minimum recommended interval of five days between reapplications. Random samples of terminal residues were taken three, five, seven, and fourteen days after the final administration of spirotetramat. A 35 mm × 20 cm soil auger was used to collect 2 kg of soil (tubifex) samples from 12 sites per plot at a depth of 0–15 cm.

## 5. Conclusions

In this work, the straightforward and precise approach for analyzing spirotetramat enantiomers in tubifex produced good results both qualitatively and quantitatively. We looked at the terminal residues and dissipation of spirotetramat enantiomers in tubifex. Dissipation and terminal residue data collected in 2024 revealed that the half-lives of spi-rotetramat enantiomers in tubifex were 3.81–10.58 days, respectively. At 14 days following the final spraying for the three lowest dosage levels, the terminal residues of spirotetramat enantiomers were both below the MRLs of the USA (1.0 mg/kg) and New Zealand (1.0 mg/kg). The Ministry of Agriculture in China may find these data useful in establishing maximum residue levels (MRLs) for spirotetramat enantiomers in tubifex as well as in recommending safe and appropriate usage of these compounds in the ecosystem.

## Figures and Tables

**Table 1:** Calibration equations, correlation coefficients (R^2^), limits of detection (LODs), limits of quantitation (LOQs), and matrix effects (% ME) of spirotetramat enantiomers.

Analyte	Regression Equation	R^2^	LOQ (mg/kg)	LOD (mg/kg)	% ME
Acetone	y = 18,588x + 3866	0.9999	0.01	0.003	–
Spirotetramat enantiomers 1	y = 88,427x − 11,846	0.9999	0.01	0.003	3.8
Spirotetramat enantiomers 2	y = 193,850x + 18,167	0.9999	0.01	0.003	9.4
Spirotetramat enantiomers 3	y = 264,791x − 28,352	0.9999	0.01	0.003	0.06
Spirotetramat enantiomers 4	y = 578,364x − 82,564	0.9999	0.01	0.003	1.04

**Table 2:** Recovery and RSD values obtained for Spirotetramat enantiomers in tubifex.

Pesticide	Spike Level (mg/kg)	Recovery					Average Recoveries	RSD
Spirotetramat enantiomers 1	0.05	0.83	0.89	0.93	0.94	0.86	0.89	5.10
	1.0	1.00	0.94	1.00	0.88	0.97	0.96	5.19
	5.0	0.87	0.82	0.81	0.83	0.84	0.83	2.58
Spirotetramat enantiomers 2	0.05	1.04	1.15	1.04	0.97	0.82	1.00	2.01
	1.0	0.86	0.89	0.83	0.89	0.90	0.87	3.50
	5.0	0.87	0.89	0.86	0.98	0.88	0.89	5.29
Spirotetramat enantiomers 3	0.05	1.12	1.11	1.02	1.01	1.04	1.06	5.09
	1.0	0.83	0.93	0.87	0.97	0.98	0.91	6.87
	5.0	0.84	0.85	0.83	0.86	0.87	0.85	2.03
Spirotetramat enantiomers 4	0.05	0.91	0.92	0.88	0.86	0.89	0.89	2.57
	1.0	0.91	0.96	0.94	0.96	0.91	0.94	2.58
	5.0	0.85	0.90	0.84	0.97	0.89	0.89	5.97

**Table 3:** Degradation data of Spirotetramat enantiomers in tubifex.

Time (d)	Spirotetramat Enantiomers 1		Spirotetramat Enantiomers 2		Spirotetramat Enantiomers 3		Spirotetramat Enantiomers 4	
	Residue (mg/kg)	Degradation Rate (%)	Residue (mg/kg)	Degradation Rate (%)	Residue (mg/kg)	Degradation Rate (%)	Residue (mg/kg)	Degradation Rate (%)
0	0.65	/	0.72	/	0.31	/	0.54	/
1	0.38	41.54	0.36	50.00	0.27	12.90	0.49	9.26
3	0.17	73.85	0.23	68.06	0.25	19.35	0.46	14.81
5	0.16	75.38	0.15	79.17	0.23	25.81	0.40	25.93
7	0.15	76.92	0.12	83.33	0.15	51.61	0.26	51.85
10	0.15	76.92	0.11	84.72	0.04	87.10	0.14	74.07
14	0.11	83.08	ND	/	ND	/	0.13	75.93
21	0.09	86.15	ND	/	ND	/	0.09	83.33
28	0.06	90.77	ND	/	ND	/	0.08	85.19
Equation	C = 0.3150e^−0.0655t^		C = 0.4773e^−0.1772t^		C = 0.3756e^−0.1821t^		C = 0.4823e^−0.0758t^	
R	−0.8657		−0.9202		−0.9003		−0.9445	
T_1/2_ (d)	10.58		3.90		3.81		9.14	

**Table 4:** Terminal residues of Spirotetramat enantiomers in tubifex in China in 2024.

Dosage	667 Times Liquid						1000 Times Liquid					
Number of applications	3			4			3			4		
Pre-harvest interval (d)	3	5	7	3	5	7	3	5	7	3	5	7
Residue (mg/kg)												
enantiomers 1	0.54 ± 0.03	0.42 ± 0.05	0.20 ± 0.01	0.66 ± 0.02	0.59 ± 0.07	0.21 ± 0.01	1.13 ± 0.08	0.92 ± 0.07	0.03 ± 0.01	0.83 ± 0.07	0.64 ± 0.05	0.31 ± 0.03
enantiomers 2	0.49 ± 0.02	0.22 ± 0.02	0.18 ± 0.05	0.54 ± 0.08	0.41 ± 0.06	0.29 ± 0.02	0.42 ± 0.06	0.32 ± 0.04	0.19 ± 0.03	0.68 ± 0.06	0.42 ± 0.06	0.25 ± 0.01
enantiomers 3	0.49 ± 0.04	0.11 ± 0.01	0.09 ± 0.01	0.46 ± 0.06	0.33 ± 0.08	0.11 ± 0.01	0.25 ± 0.04	0.22 ± 0.03	0.07 ± 0.01	0.52 ± 0.05	0.39 ± 0.02	0.12 ± 0.01
enantiomers 4	0.39 ± 0.02	0.30 ± 0.01	0.23 ± 0.01	0.52 ± 0.07	0.41 ± 0.03	0.30 ± 0.03	0.95 ± 0.08	0.39 ± 0.03	0.21 ± 0.01	0.97 ± 0.08	0.64 ± 0.07	0.22 ± 0.02

## Data Availability

The original contributions presented in this study are included in the article. Further inquiries can be directed to the corresponding authors.
